# Navigating Diagnostic Challenges: Severe Pulmonary Hypertension in Acute Exacerbation of Chronic Obstructive Pulmonary Disease vs. Pulmonary Embolism

**DOI:** 10.7759/cureus.56907

**Published:** 2024-03-25

**Authors:** Ammar Abdulfattah, Sabu John

**Affiliations:** 1 Department of Internal Medicine, State University of New York Downstate Medical Center, Brooklyn, USA; 2 Department of Cardiology, State University of New York Downstate Medical Center, Brooklyn, USA

**Keywords:** pocus, echocardiography, right heart strain, pulmonary embolism, pulmonary hypertension

## Abstract

A 63-year-old male with an unremarkable medical history presented to the emergency room (ER) with shortness of breath and bilateral lower extremity edema. In the ER, he was found to be hypoxic and hypercapnic on an arterial blood gas. CT angiography of the chest revealed severe emphysematous changes and large right apical bullae. A bedside point-of-care ultrasound demonstrated many bilateral B-lines as well as normal ejection fraction (EF). An echocardiogram revealed a small left ventricular cavity with an EF of 65%, severely dilated right ventricle, severe right ventricular dysfunction, "D" shaped interventricular septum, severely dilated right atrium, and severe pulmonary arterial hypertension (PAH) with a calculated pulmonary artery systolic pressure of 72 mmHg. The patient was initiated on bilevel positive airway pressure, glucocorticoids, bronchodilator nebulization, and diuretics with symptomatic improvement. Herein, this case report discusses similarities and differences between presentations and echocardiographic manifestations of severe PAH in the setting of acute exacerbation of chronic obstructive pulmonary disease and pulmonary embolism in the acute setting.

## Introduction

Acute hypoxic and hypercapnic respiratory failure secondary to end-stage chronic obstructive pulmonary disease (COPD) with severe pulmonary arterial hypertension (PAH) and acute pulmonary embolism (PE) are two medical emergencies with high morbidity and mortality rates [1]. Therefore, it is of paramount importance to be able to differentiate and delineate both diagnoses so as to treat them promptly. Interestingly, both entities share many similarities, clinically and in echocardiographic findings [2]. The phrase “D-sign” or D-shaped interventricular septum is suggestive of right ventricular pressure and volume overload on an echocardiogram and has almost become synonymous with pulmonary embolism in the acute clinical setting. However, it is crucial to remember that other pathologies that increase the pulmonary artery and right heart pressures can lead to the D-sign in the acute setting [2]. Our patient presented with symptoms and signs such as hypoxic respiratory failure, acute worsening of shortness of breath, tachycardia, and D-sign on point-of-care-ultrasound (POCUS) which can be confused for PE at first glance. Nevertheless, the same can be said about cor pulmonale, defined as right ventricular hypertrophy and dilatation secondary to PAH caused by respiratory disorders such as COPD.

## Case presentation

A 63-year-old man, whose medical history showed no significant issues, arrived at the emergency room (ER) experiencing shortness of breath and swelling in both lower extremities. The patient reported that he had a longstanding history of shortness of breath for many years and reported lower extremity edema for two months. He was previously prescribed inhalers and Montelukast for his “shortness of breath”. He endorsed heavy marijuana use daily but denied tobacco use. Additionally, he suffered from an occasional nonproductive cough at baseline. Of note, the patient never received an official pulmonary function test (PFT) and had not seen a doctor in many years.

**Figure 1:**
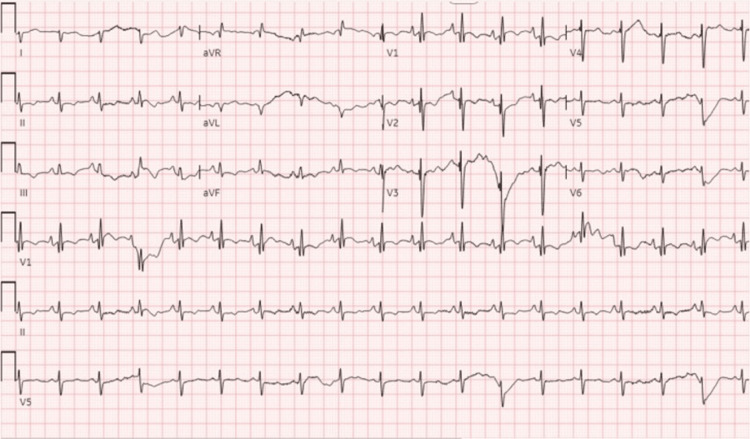
Electrocardiogram (EKG) showing sinus tachycardia at a rate of 110 with a right axis deviation, right ventricular hypertrophy, and pulmonary disease pattern

Upon presentation, the patient was noted to be hypoxic to 70% on room air and using accessory muscles for respiration, hypotensive to 93/56 mmHg, and tachycardic to 112 beats per minute (bpm). An electrocardiogram revealed sinus tachycardia at a rate of 110 with a right axis deviation, right ventricular hypertrophy, and pulmonary disease pattern (Figure 1). Arterial blood gas (ABG) was significant for a pH of 7.12, pO_2_ of 56, and pCO_2_ of 117, concurrent with severe respiratory acidosis. Other pertinent labs included an elevated pro-brain natriuretic peptide (pro-BNP) of 5153pg/ml (normal value <100pg/ml in patients under 75 years) and high-sensitivity troponin of 24ng/L (normal value <15ng/L) which down-trended on repeat (Table 1).

**Table 1:** Patient's pertinent laboratory findings

Laboratory Investigation	Result	Reference Range
Arterial Blood Gas (ABG)	-	-
- pH	7.12	7.35 - 7.45
- pO_2_ (partial pressure of O_2_)	56	75 - 100 mmHg
- pCO_2_ (partial pressure of CO_2_)	117	35 - 45 mmHg
Pro-Brain Natriuretic Peptide (pro-BNP)	5153 pg/ml	<100 pg/ml (patients under 75 years) |
High-Sensitivity Troponin	24 ng/L	<15 ng/L

**Figure 2:**
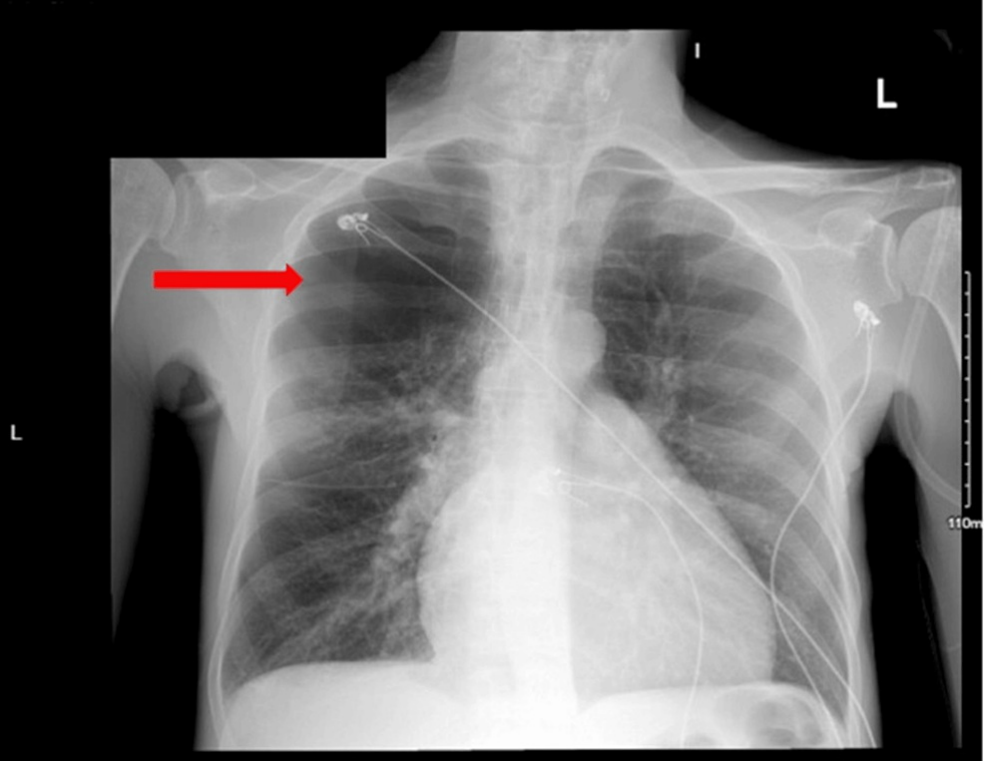
Chest X-ray of the patient showing a large right apical bulla (red arrow), prominence of the main pulmonary artery, and emphysematous changes in the lungs

**Figure 3:**
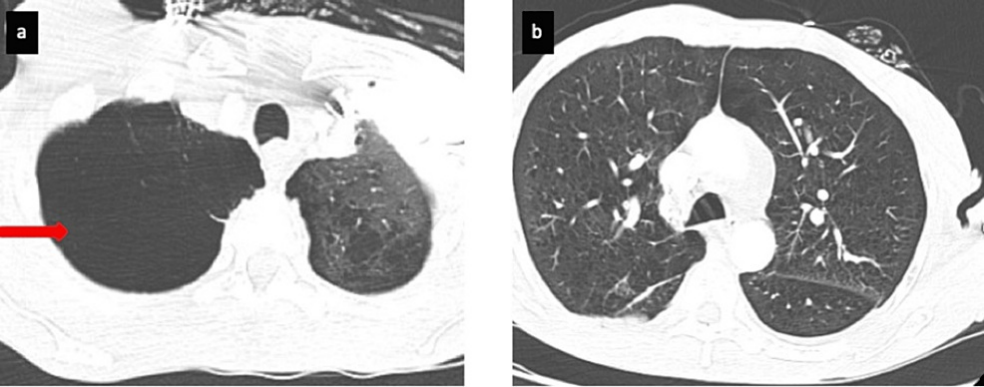
Lung window of computed tomography pulmonary angiography (CTPA) scan showing (a) large right apical lung bulla (red arrow) and (b) emphysematous changes in both lungs

A bedside POCUS in the ER demonstrated right ventricular dilation with D-sign, visually estimated normal ejection fraction (EF), and many B-lines in both lungs. A chest X-ray showed a large right apical bulla with pleural-parenchymal changes and increased atelectasis of the adjacent lung, prominence of the main pulmonary artery, and emphysematous changes in the lungs (Figure 2). CT angiography of the chest ruled out pulmonary embolism but confirmed the X-ray finding of a large right apical bulla and extensive emphysematous changes in both lungs (Figure 3). 

**Figure 4:**
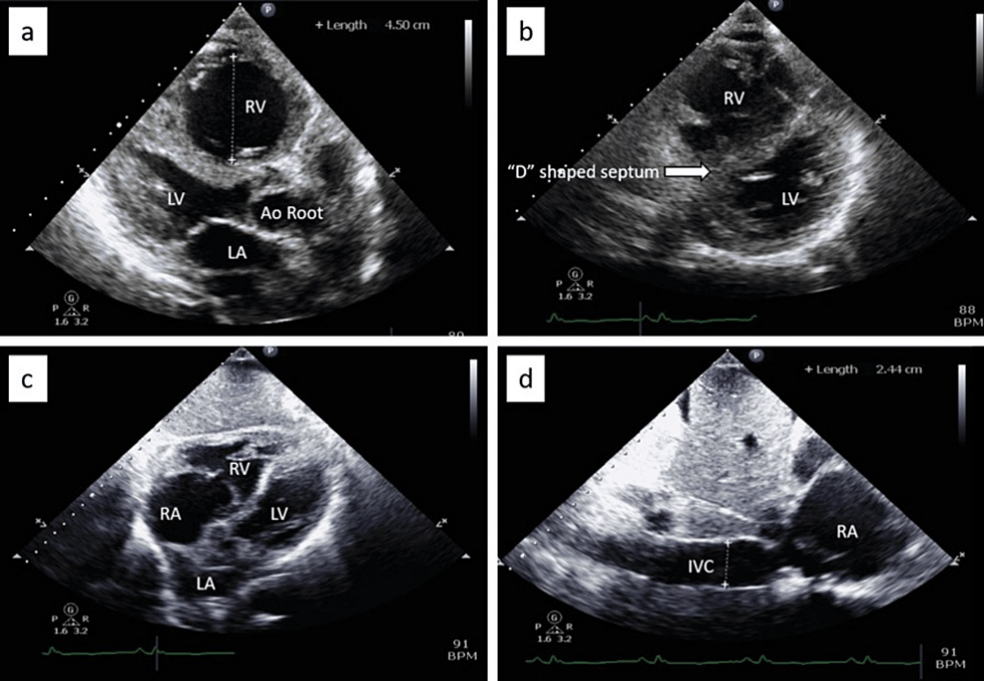
Transthoracic echocardiography (TTE) images showing the following: (a) parasternal long axis view showing a severely dilated RV measuring 4.5cm, (b) parasternal short axis view showing a “D” shaped interventricular septum sign suggestive of RV pressure and volume overload, (c) subcostal four-chamber view showing dilated RV and RA, and (d) subcostal view showing dilatation of the IVC at 2.44cm. RV: Right ventricle; LV: left ventricle; LA: left atrium; Ao root: aortic root; RA: right atrium; LA: left atrium; IVC: inferior vena cava

**Figure 5:**
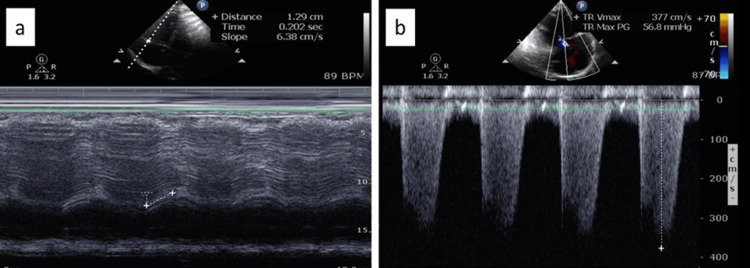
Transthoracic echocardiography (TTE) images showing the following: (a) right ventricular function measurement by tricuspid annular plane systolic excursion (TAPSE) low at 1.29cm (normal >1.7cm) and (b) tricuspid regurgitation velocity of 3.77cm/s. Calculated pulmonary artery systolic pressure (PASP) is severely elevated at 71.85mmHg PASP: Peak velocity square X 4 plus RA pressure; in this case: 3.77 squared X 4 plus 15 mmHg=71.85mmHg

An echocardiogram during hospitalization revealed a small left ventricular cavity with an EF of 65%, severely dilated right ventricle, severe right ventricular dysfunction, "D" shaped interventricular septum suggestive of right ventricular pressure and volume overload, severely dilated right atrium, and severe PAH with a calculated pulmonary artery systolic pressure (PASP) of 72 mmHg (Figure 4 and Figure 5).

The patient was immediately initiated on bilevel positive airway pressure (BiPAP), systemic glucocorticoids, bronchodilator nebulization, and diuresis with an initial improvement in pH and pCO_2_ on repeat venous blood gas to 7.18 and 94mmHg, respectively. The patient was admitted for acute hypoxic hypercapnic respiratory failure secondary to acute exacerbation of COPD (AECOPD) where he continued to improve on BiPAP and was later switched to nasal cannula O_2_ therapy. When the patient was clinically improved, he was discharged home with BiPAP at night and close follow-up with pulmonology and referrals for a sleep study and PFT.

## Discussion

In an acute setting, the main goal in the management of a patient in acute hypoxic hypercapnic respiratory failure is to narrow down the differential diagnosis as well as start emergent treatment in lieu of acute catastrophic progression of the disease. The treatment pathways for AECOPD with acute worsening of right heart failure could not be more different from that of massive pulmonary embolism with right heart strain. Therefore, it is of crucial importance to differentiate the two as early as possible. Both disease processes share common signs and symptoms including dyspnea, tachypnea, tachycardia, rales, decreased breath sounds, cough, pleuritic chest pain, fevers, and lower extremity swelling [3-5]. ABG findings in both AECOPD and PE can include hypoxemia. Hypercapnia may be noted in AECOPD and also in advanced respiratory failure secondary to PE [3,6]. Since PE manifestations can appear similar to AECOPD, it is often difficult to distinguish between the two conditions. Moreover, patients with COPD carry approximately twice the risk of developing PE than those without COPD, which makes differentiating the two even more challenging, and cor pulmonale was found to be an independent risk factor for PE [7,8]. Given the shared echocardiographic findings of right ventricular dysfunction in both conditions, there is a risk of unintended misdiagnosis, improper and potentially detrimental treatment, and delays in administering time-critical therapies. Hence, it becomes imperative to assess the efficacy of echocardiography in diagnosing and differentiating these conditions accurately and promptly.

Doppler echocardiography is the non-invasive method of choice for the diagnosis of PAH [9,10], which can estimate the systolic and diastolic pulmonary artery pressures by measuring the maximum velocity of the tricuspid and pulmonary valve regurgitation using continuous wave Doppler echocardiography respectively, as well as allow an assessment of cardiac structure and function. The 2022 European Society of Cardiology (ESC) and European Respiratory Society (ERS) guidelines recommended echocardiography as the first-line, non-invasive diagnostic test for PAH. In addition, approximately 30 to 40 percent of patients with PE have echocardiographic abnormalities indicative of right ventricular (RV) strain or pressure overload [11,12]. RV strain on echocardiography, which can be seen in PAH and PE, can be visualized as a combination of multiple findings (Figure 4 and Figure 5). These include tricuspid regurgitation, increased right ventricle: left ventricle size ratio, elevated PASP, decreased TAPSE, abnormal septal motion, McConnell’s sign (regional wall motion abnormalities that spare the right ventricular apex), 60/60 sign (the coexistence of a truncated right ventricular outflow tract acceleration time (AT <60 ms) with a PASP of less than 60 mmHg, but more than 30 mmHg), decreased S’ (lateral tricuspid annulus peak systolic velocity), pulmonary artery mid-systolic notching, and speckle tracking demonstrating decreased RV free wall strain [11-13].

Some of these parameters can help distinguish between acute or chronic RV dysfunction in PE and PAH, respectively. Those suggestive of acute RV dysfunction include a right heart thrombus, RV free wall thickness which is equal to or less than 5mm, tricuspid regurgitation pressure gradient equal to or less than 46mmHg, which corresponds to tricuspid regurgitation maximal velocity equal to or less than 3.4 m/sec, 60/60 sign, pulmonary artery early-systolic notching, pulmonary artery acceleration time 60-80 msec, McConnell’s sign, right atrial enlargement which is equal to the size of the left atrium, and pulmonary artery early-systolic notching [14]. On the other hand, RV free wall thickness of more than 5mm, tricuspid regurgitation pressure gradient of more than 46mmHg corresponding to tricuspid regurgitation maximal velocity of more than 3.4 m/sec, pulmonary artery acceleration time of more than 105 msec, and right atrial enlargement greater than the left atrial size all suggest chronic RV dysfunction (Table 2) [14]. Regional wall motion abnormalities, specifically those sparing the RV apex also known as the McConnell sign, exhibit a sensitivity of 77 percent in diagnosing PE. However, this sign can serve as a distinguishing factor in patients displaying it, helping differentiate between individuals experiencing RV strain due to acute PE and those with PAH. The latter group typically presents with a broader spectrum of global RV dysfunction [15].

**Table 2:** Echocardiographic findings in acute and chronic right ventricular (RV) dysfunction in pulmonary embolism (PE) and pulmonary arterial hypertension (PAH), respectively

Parameter	Acute RV Dysfunction	Chronic RV Dysfunction
Right heart thrombus	Present	Absent
Right ventricular free wall thickness	≤ 5mm	> 5mm
Tricuspid regurgitation pressure gradient	≤ 46mmHg (TR max velocity ≤ 3.4 m/sec)	> 46mmHg (TR max velocity > 3.4 m/sec)
60/60 sign	Present	Absent
Pulmonary artery early-systolic notching	Present	Absent
Pulmonary artery acceleration time	60-80 msec	> 105 msec
McConnell’s sign	Present	Absent
Right atrial enlargement	Equal to the size of the left atrium	Greater than the size of the left atrium

## Conclusions

In the appropriate clinical setting, identifying echocardiographic indicators of acute right ventricular dysfunction can serve as a valuable indicator for diagnosing PE and hastening the use of definitive imaging, such as computed tomography pulmonary angiography (CTPA). In cases where a patient is hemodynamically unstable or CTPA is delayed or inaccessible, these findings might prompt the initiation of anticoagulant therapy or thrombolytics. Conversely, detecting echocardiographic markers suggestive of chronic RV dysfunction can signal the presence of PAH. Recognizing PAH is crucial for implementing suitable resuscitative measures.

## References

[1] Beshay S, Sahay S, Humbert M (2020). Evaluation and management of pulmonary arterial hypertension. Respir Med.

[2] Fields JM, Davis J, Girson L (2017). Transthoracic echocardiography for diagnosing pulmonary embolism: a systematic review and meta-analysis. J Am Soc Echocardiogr.

[3] Stein PD, Terrin ML, Hales CA, Palevsky HI, Saltzman HA, Thompson BT, Weg JG (1991). Clinical, laboratory, roentgenographic, and electrocardiographic findings in patients with acute pulmonary embolism and no pre-existing cardiac or pulmonary disease. Chest.

[4] Stein PD, Beemath A, Matta F (2007). Clinical characteristics of patients with acute pulmonary embolism: data from PIOPED II. Am J Med.

[5] Liu X, Jiao X, Gong X (2023). Prevalence, risk factor and clinical characteristics of venous thrombus embolism in patients with acute exacerbation of COPD: a prospective multicenter study. Int J Chron Obstruct Pulmon Dis.

[6] Sansone EB, Losikoff AM, Pendleton RA (1977). Potential hazards from feeding test chemicals in carcinogen bioassay research. Toxicol Appl Pharmacol.

[7] Keramidas G, Gourgoulianis KI, Kotsiou OS (2021). Venous thromboembolic disease in chronic inflammatory lung diseases: knowns and unknowns. J Clin Med.

[8] Cao YQ, Dong LX, Cao J (2018). Pulmonary embolism in patients with acute exacerbation of chronic obstructive pulmonary disease. Chin Med J (Engl).

[9] Naeije R, Torbicki A (1995). More on the noninvasive diagnosis of pulmonary hypertension: Doppler echocardiography revisited. Eur Respir J.

[10] McGoon M, Gutterman D, Steen V, Barst R, McCrory DC, Fortin TA, Loyd JE (2004). Screening, early detection, and diagnosis of pulmonary arterial hypertension: ACCP evidence-based clinical practice guidelines. Chest.

[11] Gibson NS, Sohne M, Buller HR (2005). Prognostic value of echocardiography and spiral computed tomography in patients with pulmonary embolism. Curr Opin Pulm Med.

[12] Kucher N, Rossi E, De Rosa M, Goldhaber SZ (2005). Prognostic role of echocardiography among patients with acute pulmonary embolism and a systolic arterial pressure of 90 mm Hg or higher. Arch Intern Med.

[13] Alerhand S, Sundaram T, Gottlieb M (2021). What are the echocardiographic findings of acute right ventricular strain that suggest pulmonary embolism?. Anaesth Crit Care Pain Med.

[14] Alerhand S, Adrian RJ (2023). What echocardiographic findings differentiate acute pulmonary embolism and chronic pulmonary hypertension?. Am J Emerg Med.

[15] McConnell MV, Solomon SD, Rayan ME, Come PC, Goldhaber SZ, Lee RT (1996). Regional right ventricular dysfunction detected by echocardiography in acute pulmonary embolism. Am J Cardiol.

